# Impact of Human Activities and Climate Change on Chinese Forest Musk Deer (*Moschus berezovskii*)

**DOI:** 10.3390/biology15070549

**Published:** 2026-03-30

**Authors:** Du Xu, An-Bang Cui, Xu-Lu Ming, Yu-Lu Fei, Xue-Rui Yang, Wen-Bo Li

**Affiliations:** 1 School of Resources and Environmental Engineering, Anhui University, Hefei 230601, China; xd606638@163.com (D.X.); c3277902316@163.com (A.-B.C.); 2 International Collaborative Research Center for Huangshan Biodiversity and Tibetan Macaque Behavioral Ecology, Hefei 230601, China; m152540x@163.com (X.-L.M.); 17856536960@163.com (Y.-L.F.); 3 School of Life Sciences, Anhui University, Hefei 230601, China

**Keywords:** human activities, climate change, forest musk deer, species distribution model, habitat suitability

## Abstract

Human activities and climate change are influencing the survival and distribution of species, threatening the current distribution pattern of biodiversity and potentially leading to the “sixth mass extinction.” The forest musk deer (*Moschus berezovskii*) is among the most numerous and widely distributed musk deer species in China. However, its habitat is severely threatened by human activities and climate change. Due to the lack of field surveys and research data, it is difficult to assess the threats posed by human activities and climate change effectively. In this study, we integrate the new records of forest musk deer with climate and human activity data, and apply the MaxEnt species distribution model to evaluate the impact of human activities and climate change on the forest musk deer under current conditions and future scenarios (SSP1-2.6 and SSP5-8.5 for the 2030s, 2050s, and 2070s). Our results showed that the forest musk deer prefer areas with high vegetation cover (NDVI > 0.7), low GDP, and low levels of human activity disturbance. The areas of high-suitability habitats are 90.10 × 10^4^ km^2^, 72.85 × 10^4^ km^2^, and 30.43 × 10^4^ km^2^, respectively. The optimal climatic conditions are an annual precipitation (BIO12) of 750–1500 mm and a seasonal temperature variation (BIO4) of 500–600. Their occurrence probability is highest at elevations between 1500 and 3000 m. Under the current climate conditions, the area of high-suitability habitats is estimated at 5.54 × 10^4^ km^2^, primarily distributed across central–northern Sichuan, northwestern Guangxi, and southern Gansu. Under the future climate scenarios, low and medium-suitability habitats are projected to shrink to varying degrees, whereas the high-suitability area is expected to expand, particularly under the SSP5-8.5-2030s scenario where it is projected to increase by 2.88 × 10^4^ km^2^. The centroid of suitable habitat is projected to shift toward higher-elevation areas in northwestern China, with regional hotspots emerging in southwestern regions such as central–northern Sichuan and northwestern Guangxi. These elevational and distributional shifts highlight the vulnerability of current habitats and the importance of adaptive conservation strategies to strengthen species protection, including continuously advancing forest protection programs, mitigating the impact of human activities in high-altitude areas, and strengthening the protection of key areas in the southwestern region.

## Simple Summary

Understanding how climate change and human activities affect wildlife habitats is essential for effective conservation. The forest musk deer is a widely distributed species in China, but its habitat is increasingly threatened by environmental change and human disturbance. In this study, we used the MaxEnt model to predict the current and future suitable habitat of forest musk deer under climate change and anthropogenic disturbance. We found that forest musk deer prefer areas with dense vegetation and less human activity, especially in mountainous regions. In the future, suitable habitats are likely to shift toward higher elevations and southwestern China, while habitats in lowland and coastal areas may shrink. These results suggest that protecting forests, limiting human disturbance, and maintaining connected habitats are important for the long-term conservation of forest musk deer.

## 1. Introduction

Biodiversity patterns and species distributions are increasingly shaped by the combined effects of environmental change and socioeconomic development [1]. Changes in land use practices, resource demand, and climatic conditions can alter habitat structure, availability, and connectivity, thereby influencing species persistence and spatial dynamics. Since the 1950s, global population growth has accelerated markedly, with annual growth rates exceeding 1.8% at certain periods. As of 15 November 2022, the United Nations announced that the global population had reached 8 billion. Population growth and economic expansion have intensified interactions between human systems and natural ecosystems, leading to widespread landscape transformation [2]. Recent assessments indicate that land use change, including agricultural expansion, urban development, and transportation infrastructure construction—has become one of the dominant drivers of terrestrial habitat modification worldwide. These processes can influence ecological vulnerability by altering both the quantity and quality of suitable habitats for many species [3]. Concurrently, climate change is reshaping temperature and precipitation regimes, thereby modifying the environmental conditions under which species persist. In response to changing climates, many species exhibit distributional adjustments along latitudinal and elevational gradients. Chen [4] reported that a wide range of terrestrial organisms, including trees, insects, and mammals, have shifted their ranges toward higher latitudes and elevations at average rates of approximately 16.9 km and 11.0 m per decade, respectively. Similarly, studies focusing on montane terrestrial animals have documented upward elevational shifts in species distributions [5]. However, the ability of species to track suitable climatic conditions is constrained by physiological tolerances, dispersal capacity, habitat fragmentation, and biotic interactions. When the pace of environmental change exceeds species’ adaptive or migratory capacity, mismatches between climatic conditions and habitat requirements may arise, potentially increasing the risk of local extinction in certain regions [6]. Across many parts of the world, rapid economic and social development has been accompanied by profound changes in land use patterns and ecosystem structure. Regions undergoing industrialization, infrastructure expansion, and urban growth often experience increased demands on natural resources, resulting in complex trade-offs between economic development and biodiversity conservation [7]. China, characterized by pronounced environmental gradients and high biodiversity, provides an informative context for examining these interactions. As the world’s second-largest economy, China has a population of approximately 1.4 billion, and its Human Development Index (HDI) ranked 82nd globally according to the IMF World Economic Outlook (2022; https://www.imf.org/, accessed on 22 July 2024). During the transition from an agricultural economy to a highly industrialized one, China’s natural environment has undergone substantial changes. For example, between 1950 and 2004, the area of natural forests declined to approximately 30% of total forest cover. By 2021, the total length of highways reached 5.2807 million km, and both highway and high-speed rail networks ranked first globally in total mileage [8]. Over recent decades, nationwide changes in land use, transportation networks, and forest management practices have continuously reshaped ecological landscapes. Understanding how wildlife responds to these transformations is essential for developing conservation strategies that are both scientifically grounded and compatible with long-term socioeconomic development. Species distribution models (SDMs) are widely used to predict changes in species distribution ranges under various climate change and socioeconomic scenarios [9,10]. In particular, a growing volume of species occurrence records and local environmental information has been utilized to develop conservation strategies for species and regions. Examples of such data include population size, GDP, vegetation cover, road construction, and protected area establishment. In recent years, researchers have increasingly employed SDMs to predict shifts in species distributions and the spatial arrangement of suitable versus unsuitable habitats based on data related to human activities and climate change [11]. However, previous studies exhibit several limitations: (1) They often focus on a limited number of species, typically a single species or a few select species. (2) The study areas are frequently confined to specific regions or individual protected areas. (3) The analysis tends to focus on a narrow set of factors, primarily emphasizing the impact of climate change on species distributions or examining only a few isolated variables. (4) The datasets used for predictions often rely on outdated climate and environmental data [11,12,13,14]. Consequently, these approaches failed to reflect the dynamics of species distribution shifts in response to diverse climate change and socioeconomic scenarios.

The forest musk deer (*Moschus berezovskii*), also known as the musk deer, belongs to the order Artiodactyla, family *Moschidae*, and genus *Moschus*. It is listed as Endangered (EN) by the International Union for Conservation of Nature (IUCN) and is a Class I nationally protected wildlife species in China. Widely distributed across central and southwestern China [15], it is also the largest extant species of musk deer in the country. The forest musk deer play an important ecological role and are also of considerable economic significance due to the medicinal value of musk secreted by adult males. Previous studies indicate that more than 70% of global musk and musk-related products originate from China [16]. Historical records suggest that wild populations experienced substantial declines during the latter half of the 20th century [17,18], a pattern associated with habitat modification, hunting pressure, and broader environmental change. Although captive breeding programs and conservation initiatives have contributed to population stabilization or recovery in some regions [19,20], the current distribution and long-term habitat suitability of the forest musk deer remain influenced by environmental conditions and patterns of human land use. In this study, we integrate occurrence records of the forest musk deer with updated climatic and anthropogenic variables to model its current and potential future habitat suitability across China. Using a MaxEnt-based species distribution framework and CMIP6 climate scenarios, our objectives are to (1) identify key environmental and socioeconomic factors associated with the species’ distribution, (2) assess potential shifts in suitable habitats under future climate conditions, and (3) provide a quantitative basis for conservation planning and adaptive management. By adopting an objective and integrative perspective, this study aims to contribute to a balanced understanding of how environmental change and human activities jointly shape species distribution patterns.

## 2. Materials and Methods

### 2.1. Occurrence Data

**Figure 1:**
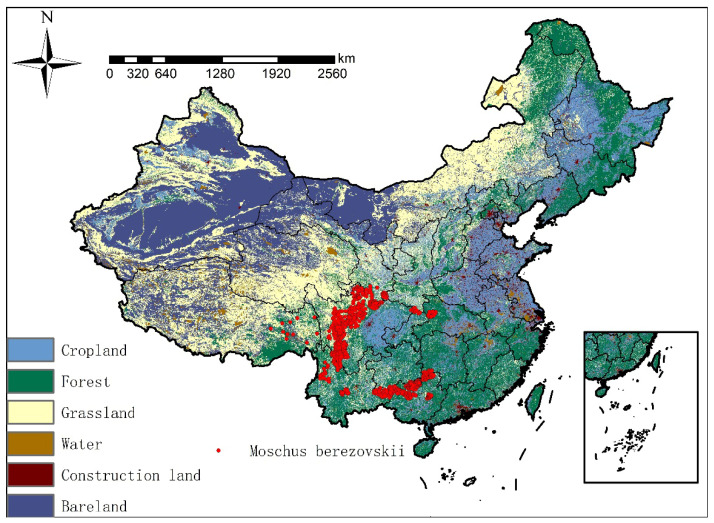
The distribution of forest musk deer in China. The red color is the occurrence data of forest musk deer. This map was based on the Chinese standard map released by the Ministry of Natural Resources in 2023, with the drawing review No. GS (2023) 2767, and the base map was not modified. The same below.

To acquire the distribution occurrence data for this study, we systematically searched and compiled relevant scientific literature, including peer-reviewed publications from domestic and international sources, as well as news reports from national newspapers, conservation magazines, and bulletins. Additionally, georeferenced occurrence records of the forest musk deer were downloaded from the Global Biodiversity Information Facility (GBIF; https://www.gbif.org; Occurrence Download https://doi.org/10.15468/dl.6tycvz (accessed on 22 July 2024)) and the IUCN Red List of Threatened Species (http://www.iucnredlist.org; Occurrence Download https://dx.doi.org/10.2305/IUCN.UK.2015-4.RLTS.T13894A61976926.en (accessed on 22 July 2024)). Searches were conducted using the keywords “forest musk deer” and “*Moschus berezovskii*”. Only records with precise geographic coordinates, collected between 1929 and 2020, were retained. Records lacking coordinates, showing obvious georeferencing errors, or falling outside the target time period were excluded. When species occurrence records in published literature solely contain locality names, georeferencing was conducted through the following steps: first, the described locations were georeferenced using the Google Earth platform; subsequently, by integrating the original occurrence data, textual descriptions of the area from the literature, and consultations with domain experts, the reported sites were verified and confirmed as the actual data collection locations. A total of 2012 current occurrence localities thus obtained constitute the initial dataset. To mitigate potential spatial autocorrelation among the points, spatial filtering was performed using ENMTools 1.4.4. Occurrence points within a specified minimum distance of 1 km of each other were filtered to retain only one representative point per grid cell or neighborhood [11]. Following this procedure, a final set of 1590 forest musk deer occurrence points was obtained (Figure 1). These data were then converted into CSV format for subsequent analysis.

### 2.2. Environmental Factors

The forest musk deer is a typical forest-dwelling species, primarily inhabiting coniferous–broadleaf mixed forests and broadleaf forests at altitudes above 1000 m [21]. National land cover data were referenced from the China Multi-period Land Use Remote Sensing Monitoring Dataset (CNLUCC) [22]. Previous studies have shown that the key environmental factors influencing the habitat selection of forest musk deer vary across different research scales. At broad spatial scales, habitat selection is at large influenced by landscape environmental factors, such as human disturbance, vegetation type, topography, and landform [23]. This study adopts a national-scale perspective to assess the distribution patterns and habitat suitability of the species across China. A total of 25 environmental predictors were incorporated, including 19 bioclimatic variables, the Normalized Difference Vegetation Index (NDVI, 2020), and the Digital Elevation Model (DEM). In addition, four anthropogenic variables were included to quantify human pressures: national population density (PD) and Gross Domestic Product (GDP), as proxies for the intensity of human settlement and economic activity; distance to roads, as a measure of infrastructure-related disturbance and habitat fragmentation; and the distribution of protected areas (PA), to represent the degree of legal protection and management. The 19 bioclimatic variables were obtained from WorldClim 2.0 (1970–2000) [24] at a resolution of 2.5 min, and future climate data were acquired for the periods 2020–2040 (2030s), 2040–2060 (2050s), and 2060–2080 (2070s). Environmental data at a 2.5 min resolution were retrieved under the Beijing Climate Center–Climate System Model version 2–Medium Resolution (BCC-CSM2-MR) of the Coupled Model Intercomparison Project Phase 6 (CMIP6). CMIP6 primarily includes seven Shared Socioeconomic Pathways (SSPs): SSP1-1.9, SSP1-2.6, SSP2-4.5, SSP3-7.0, SSP4-3.4, SSP4-6.0, and SSP5-8.5 [25]. This study employed two climate scenarios: SSP1-2.6 (a low radiative forcing scenario) and SSP5-8.5 (a high radiative forcing scenario). The Normalized Difference Vegetation Index (NDVI), Digital Elevation Model (DEM), national population density (PD), distance to roads, distribution of protected areas (PA) across China, and Gross Domestic Product (GDP) were sourced from the Resource and Environment Science Data Registration and Publishing System (https://www.resdc.cn/Default.aspx (accessed on 22 July 2024)) [26]. Among these, NDVI, DEM, PD, distance to roads, and GDP have a spatial resolution of 30 m. Based on the spatial analysis module of the ArcGIS 10.8 (ESRI) platform, the Euclidean Distance tool was used to generate a linear distance raster layer of the road network within the study area, with a spatial resolution of 30 m. The protected area (PA) variable was quantified as the Euclidean distance to the nearest protected area boundary. All protected area polygons were first projected to a uniform coordinate system consistent with other environmental layers. The polygon boundaries were then converted into raster format, after which the Euclidean Distance tool in ArcGIS 10.8 was applied to calculate the distance (in kilometers) from each grid cell to the nearest protected area. The resulting continuous raster layer was resampled to match the spatial resolution and extent of other predictor variables and subsequently converted into ASCII format for use in MaxEnt 3.4.4. All spatial data were reprojected to the GCS_WGS_1984 geographic coordinate system prior to analysis to ensure spatial alignment and prevent potential misalignment [11].

### 2.3. Data Analysis and Modeling

#### 2.3.1. Screening of Forest Musk Deer Occurrence Points and Environmental Factors Using ENMTools

**Figure 2:**
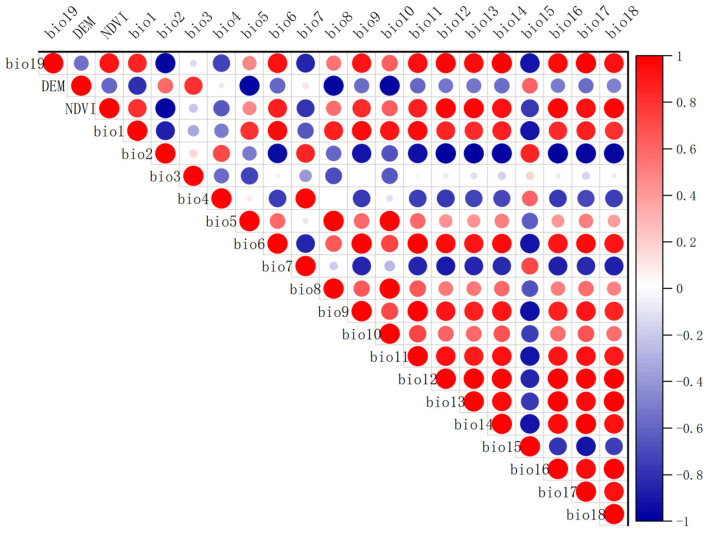
Visual results of correlation analysis of environmental factors.

Spatial autocorrelation may exist among environmental variables and occurrence points of the forest musk deer, which could lead to overfitting and compromise the accuracy of species habitat suitability predictions if the MaxEnt model is applied directly. To address this issue, this study employed Environmental Niche Models Tools (ENMTools) to screen both the species occurrence data and environmental factors. ENMTools is a software designed for quantitative niche analysis and can interact with MaxEnt, facilitating the evaluation of ecological niche models [27]. Specifically, the study employed the spatial thinning module of ENMTools to remove redundant occurrence records within the same grid cell (at a resolution of 2.5 min). Additionally, its variable correlation analysis tool was used to calculate the Pearson correlation coefficients among predictor variables (Figure 2). To preserve the ecological distinction between biophysical drivers and anthropogenic pressures, natural factors and human disturbance factors were analyzed separately. This approach ensures that the multicollinearity assessment reflects relationships within each category rather than artificially conflating effects across fundamentally different types of factors—a common practice in species distribution modeling to maintain interpretability and avoid confounding distinct ecological pathways [28]. Environmental variables exhibiting an absolute Pearson correlation coefficient greater than 0.80 were excluded, as this threshold is widely adopted in ecological modeling to mitigate high multicollinearity (corresponding to approximately 64% shared variance), thereby reducing the risk of model instability, overfitting, and biased parameter estimates—a convention supported by established literature on multivariate regression in ecology [28]. Additionally, variables with zero percent contribution to preliminary model fits were omitted. As a result, six natural factors and three anthropogenic disturbance factors were selected and converted into ASCII format compatible with MaxEnt (Table 1). Meanwhile, to minimize model overfitting and improve the robustness of future projections, the selection of future climate variables followed the same variable screening procedure applied to the current climate dataset. Specifically, climatic variables with zero percent contribution in the baseline MaxEnt model were excluded from future projections, and only variables with relatively high contribution rates were retained. This approach assumes that the relative importance of key climatic drivers remains stable across current and future scenarios and has been widely adopted in species distribution modeling studies to improve transferability and interpretability of future projections [11].

#### 2.3.2. Ecological Suitability for the Forest Musk Deer Using MaxEnt

Model Construction: This study utilized MaxEnt 3.4.4 [29] to conduct habitat suitability analysis for the forest musk deer. Species occurrence records, compiled from field surveys, published literature, and authoritative databases (GBIF and IUCN), and covering the period from 1929 to 2020, were imported into MaxEnt 3.4.4. All occurrence locations were georeferenced using Google Earth and filtered to reduce spatial autocorrelation, ensuring a consistent spatial accuracy for model calibration. These records, together with natural environmental variables and anthropogenic disturbance factors, were then used for model construction. In the model configuration, 25% of the occurrence points were designated as a random test subset, while the remaining 75% were used as the training subset. The maximum number of background points was set to 10,000, and the output format was selected as Logistic. Additionally, the options for generating response curves, performing jackknife tests of variable importance, and applying a random seed were enabled. To better account for uncertainty in model performance, the study employed a Bootstrap approach, with the final model outputs averaged over 10 replicate runs. After model execution, ASC format output files were generated and imported into ArcGIS 10.8 for spatial visualization and mapping, resulting in the habitat suitability distribution maps [9].

Model Evaluation: To assess the accuracy of the MaxEnt model predictions, this study employed the Receiver Operating Characteristic (ROC) curve, using the Area Under the Curve (AUC) value to represent prediction accuracy. The AUC value ranges from 0.5 to 1.0, with higher values indicating greater prediction performance. Studies have shown that AUC values are classified as follows: 0.5–0.6 indicates poor, 0.6–0.7 indicates moderate, 0.7–0.8 indicates fair, 0.8–0.9 indicates good, and 0.9–1.0 indicates excellent [30]. These threshold classifications have been widely applied in species distribution modeling studies of ungulates and cervids, including musk deer and related taxa, and have been shown to provide reliable assessments of model performance in mountainous and forest ecosystems of China [23]. The potential suitable habitat values for the forest musk deer under natural and anthropogenic disturbance factors range from 0 to 1. These potential habitats were reclassified into four categories: “highly suitable” (0.6–1.0), “moderately suitable” (0.4–0.6), “low suitability” (0.2–0.4), and “unsuitable” (0–0.2) [31]. Furthermore, this study utilized MaxEnt to simulate and predict the distribution of the forest musk deer under future climate scenarios (SSP1-2.6 and SSP5-8.5). The projected changes in future habitat areas were categorized into “range expansion,” “range contraction,” and “no change.” Future habitat suitability projections were conducted using climatic variables only. Anthropogenic and land-use-related variables (including NDVI, GDP, population density, and distance to roads) were excluded from future projections due to the lack of reliable long-term forecasts and the high uncertainty associated with their future dynamics at large spatial scales. This approach allows the assessment of climate-driven changes in potential habitat suitability while avoiding additional assumptions regarding future patterns of human activities [32].

To quantitatively characterize and visualize the spatial shift of the potential distribution of the forest musk deer under climate change, we further analyzed the centroid migration trajectory of its suitable habitat. Habitat suitability maps (logistic output) generated by the MaxEnt model for the current climate (baseline period: 1970–2000) and future periods (2030s, 2050s, and 2070s) under the SSP1-2.6 and SSP5-8.5 scenarios were used for this analysis. In ArcGIS 10.8, continuous suitability values (0–1) were reclassified according to a predefined threshold (high suitability ≥ 0.6) to produce binary distribution maps, where grid cells with a value of 1 represent highly suitable habitat and those with a value of 0 represent non–highly suitable areas. These binary maps were used to delineate the core potential distribution range of the forest musk deer for each period [33].

The centroid of the highly suitable habitat for each time period (current, 2030s, 2050s, and 2070s) was calculated using the “Mean Center” tool within the Spatial Statistics Tools of ArcGIS 10.8. The resulting centroid coordinates were exported as point layers and sequentially connected according to the temporal order to generate centroid migration trajectories. These trajectories were then symbolized using different colors and directional arrows and overlaid on a map of China’s administrative boundaries to visualize the spatiotemporal shift of the potential distribution centroid of the forest musk deer [11].

## 3. Results

### 3.1. Suitable Habitat of the Forest Musk Deer

**Figure 3:**
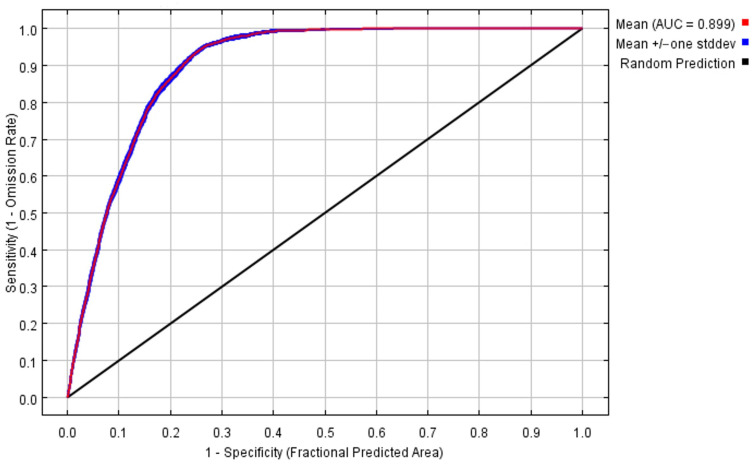
Receiver operating characteristic curve of MaxEnt model.

As shown in Figure 3, the mean AUC value of the model was 0.899 ± 0.001, indicating an excellent performance level. This result demonstrates high predictive accuracy, confirming that the model outputs reliably reflect the habitat distribution of the forest musk deer. In addition to AUC, model robustness was supported by the consistency of prediction outputs across bootstrap replicates, as well as by the response curves and jackknife tests, which together provide complementary information on model stability and the relative importance of predictor variables [11].

**Figure 4:**
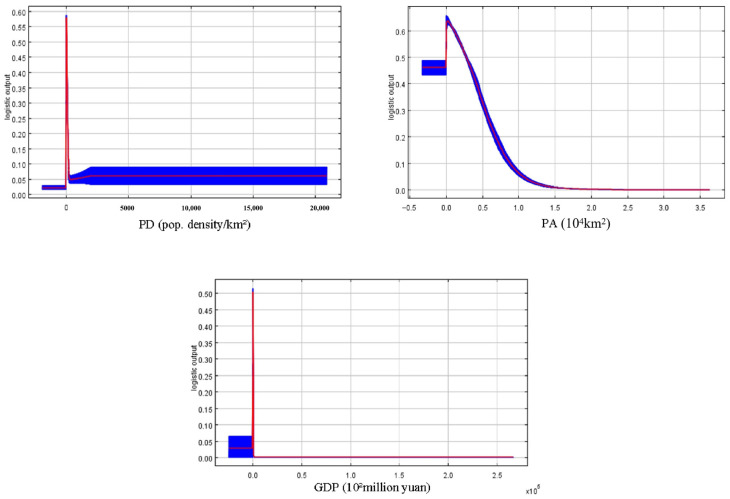
Response curves of three kinds of human interference factors.

**Figure 5:**
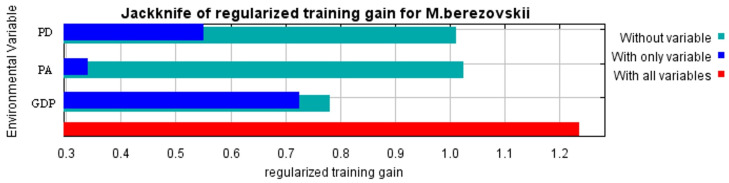
Results of knife-cutting test of environment variables.

Percent contribution, which reflects the importance of environmental variables to species distribution during MaxEnt model training, indicated that NDVI had the highest contribution (48.8%) in this study, followed by GDP (34.6%) and population density (16.6%). According to Figure 4, an NDVI value greater than 0.8 correspond to the most suitable survival range (habitat suitability > 0.6) for the forest musk deer, i.e., areas with lush vegetation. As shown in the jackknife plot (Figure 5), population density had less influence on the distribution of the forest musk deer comparing to two other variables. Additionally, the probability of forest musk deer occurrence increases with decreasing GDP values (habitat suitability > 0.5) and decreasing population density (habitat suitability > 0.55).

**Figure 6:**
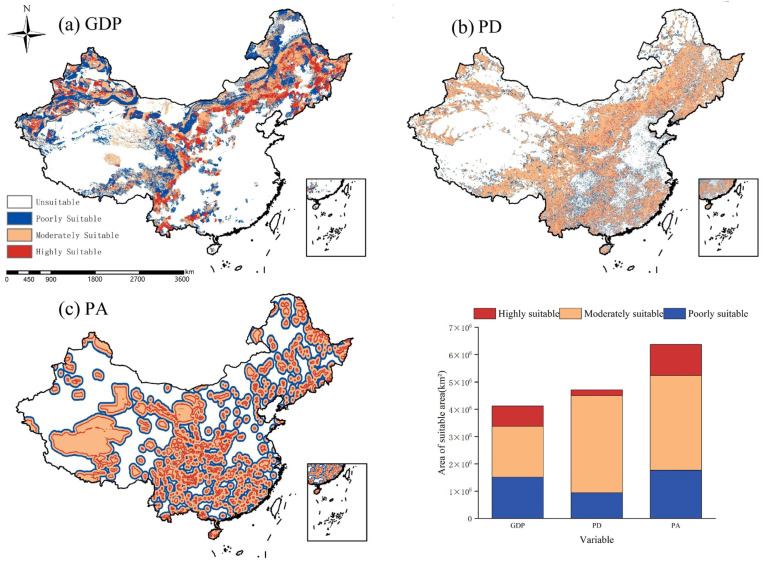
Potential distribution area of forest musk deer under different human disturbance factors.

This study selected these three anthropogenic factors (PA, GDP, and PD) that significantly influence the distribution of the forest musk deer population. Using each as the sole environmental variable, and without considering the effects of climatic, topographic, and other environmental factors on the species’ distribution, the MaxEnt model was employed to predict the potential distribution area of the forest musk deer (Figure 6).

Research results indicate that under the influence of NDVI, the highly suitable habitat for forest musk deer covers approximately 901,000 km^2^, primarily distributed across southern China. These areas include Yunnan, Hunan, Guizhou, and Guangdong Provinces; the Guangxi Zhuang Autonomous Region; Jiangxi, Fujian, and Zhejiang Provinces; the western part of Hubei Province; and the southern part of Anhui Province. The moderately suitable area encompasses approximately 940,800 km^2^, mainly located in central Sichuan Province, Chongqing Municipality, eastern Hubei Province, southwestern Anhui Province, southern Shaanxi Province, the border region between southern Shaanxi and Gansu Provinces, and the junction between Henan and Hubei Provinces.

With respect to GDP, the highly suitable area is approximately 728,500 km^2^, concentrated in northern and southern Yunnan Province, northern Guangxi Zhuang Autonomous Region, central and northern Sichuan Province, most of Gansu Province, the border region between Qinghai and Gansu Provinces, and western Shanxi Province. The moderately suitable area spans about 1,727,800 km^2^, covering central Guangxi Zhuang Autonomous Region, central and northern Yunnan Province, the border between the Tibet Autonomous Region and Sichuan Province, central and western Sichuan Province, Gansu Province, and parts of Shaanxi Province.

Under the influence of population density, the highly suitable area measures approximately 304,300 km^2^, while the moderately suitable area reaches 3,438,100 km^2^. These areas are primarily located across Yunnan Province, the Guangxi Zhuang Autonomous Region, northern Guangdong Province, most of Fujian Province, the Tibet Autonomous Region, the border between Qinghai and Sichuan Provinces, central and western Sichuan Province, eastern and southern Guizhou Province, western Hunan Province, most of Jiangxi Province, southern Anhui Province, western Zhejiang Province, western Hubei Province, eastern Chongqing Municipality, most of Gansu Province, southern Shaanxi Province, and the border region of Shaanxi, Shanxi, and Henan Provinces.

### 3.2. Suitable Habitat Under Current Climate

**Figure 7:**
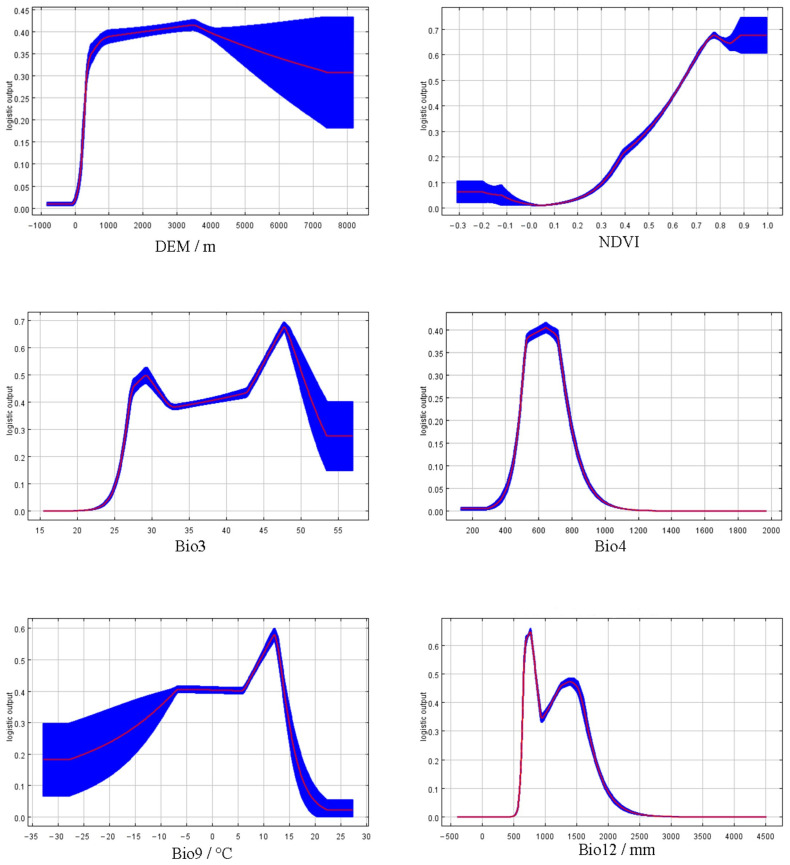
Corresponding curves of six natural factors.

As shown in Table 2, among the six natural factors, Annual Precipitation (BIO12), Temperature Seasonality (BIO4), and DEM (Digital Elevation Model) exhibited relatively higher contribution rates, at 43.5%, 32.3%, and 13.3%, respectively. Figure 7 indicates that the optimal survival range for the forest musk deer corresponds to an Annual Precipitation (BIO12) of approximately 750–1500 mm. The maximum probability of occurrence was observed at DEM values between 1500 and 3000 m. Furthermore, habitat suitability for the forest musk deer is relatively high when Temperature Seasonality (BIO4) reaches 500–600.

**Figure 8:**
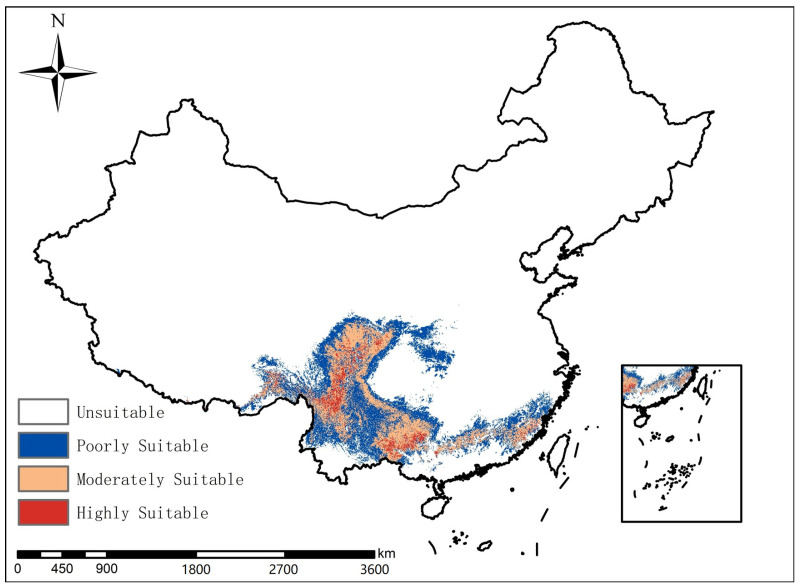
Potential habitat areas of forest musk deer in China.

As shown in Figure 8, the unsuitable area for the forest musk deer population is 853.11 × 10^4^ km^2^. The area of low suitability is 56.07 × 10^4^ km^2^, accounting for 51.29% of the total suitable area. It is primarily distributed in eastern Tibet, eastern Qinghai, southern Gansu, central–western Sichuan, central Yunnan, southern Guizhou, northwestern Guangxi, western Hunan, northern Guangdong, western Hubei, and southern Shaanxi, among other regions. The area of moderate suitability is 47.70 × 10^4^ km^2^, accounting for 43.64% of the total suitable area, mainly located in northern Yunnan, central Sichuan, most of Guizhou, northern Guangxi, southern Gansu, and southern Hunan, among other regions. The area of high suitability is 5.54 × 10^4^ km^2^, accounting for 5.07% of the total suitable area, and is primarily distributed across multiple regions including central–northern Sichuan, northwestern Guangxi, and southern Gansu.

### 3.3. Suitable Habitat Under Future Climate

**Figure 9:**
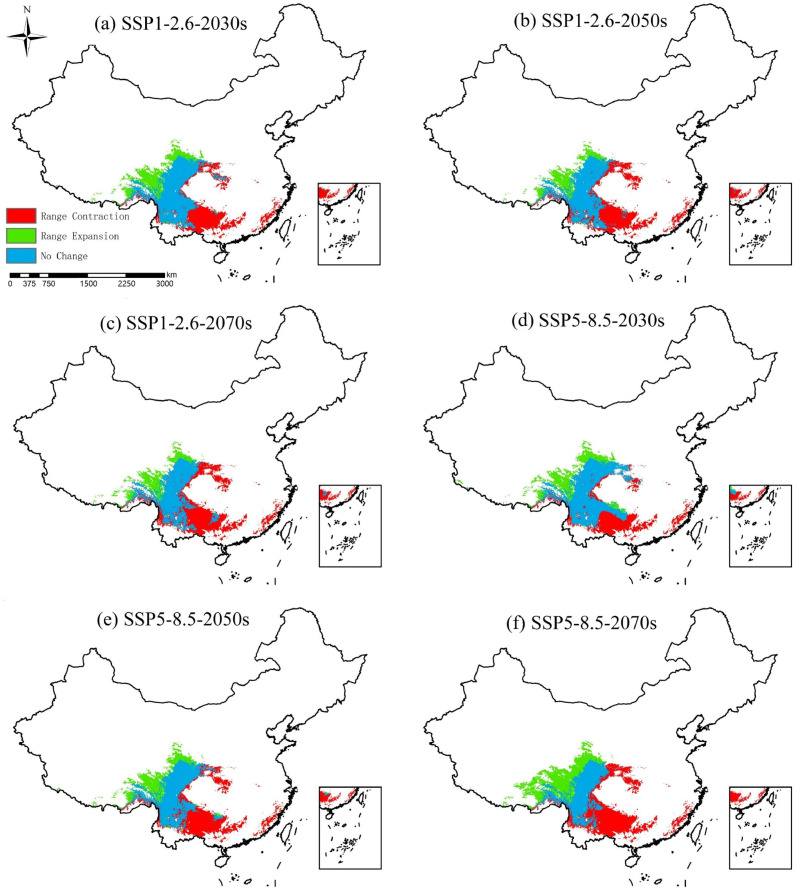
Spatial change pattern of forest musk deer’s future habitat.

Based on the future climate projections generated using the selected high-contribution climatic variables under CMIP6 scenarios, the area of suitable habitat for the forest musk deer is projected to decrease under future climate conditions (Figure 9). Among the suitability categories, moderately suitable area exhibits the most substantial reduction, declining by 9.09–28.29 × 10^4^ km^2^, with the greatest loss occurring under the SSP1-2.6-2070s scenario. The low suitability area is projected to decrease by 3.91–17.01 × 10^4^ km^2^. In contrast, the highly suitable area remains nearly unchanged or shows a slight increase, expanding by 3.68 × 10^4^ km^2^ under the SSP5-8.5-2070s scenario (Table 3). The future distribution of the forest musk deer is expected to shift towards the border region of Sichuan, Qinghai, and Tibet. Suitable habitat is projected to predominantly occupy large parts of Sichuan Province and northern Yunnan Province, with persistent and stable distribution also present in areas such as Gansu, Shaanxi, and Hubei. However, habitats in coastal regions are projected to contract significantly. During the 2030s period, the forest musk deer shows extensive distribution across most of Yunnan Province and western Guizhou Province. By the 2050s and 2070s periods, its distribution in Yunnan and Guizhou begins to contract, with notably fewer occurrences within Guizhou Province. It should be noted that local land use changes and anthropogenic disturbances, which were not incorporated into future projections, may further modify habitat availability at finer spatial scales.

**Figure 10:**
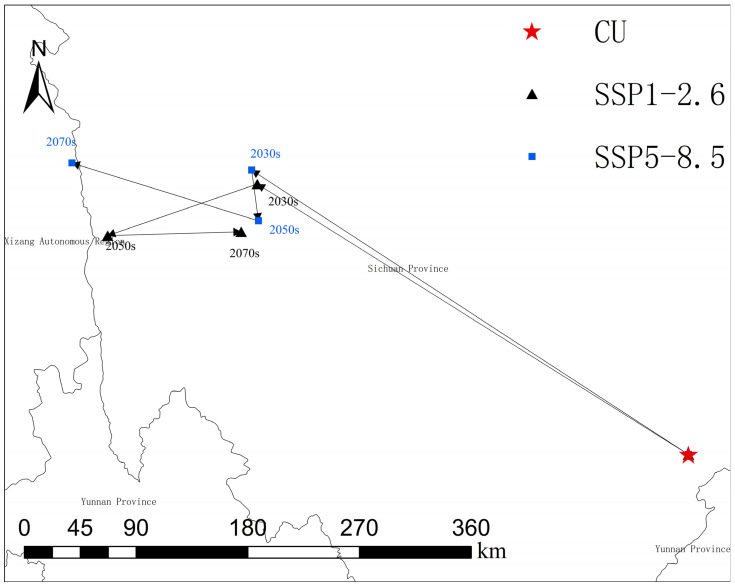
Centroid migration map of suitable habitat for forest musk deer.

As shown in Figure 10, under all future climate scenarios, the centroid of suitable habitat for the forest musk deer exhibits a pronounced northwestward shift, migrating from the current border region between Sichuan and Yunnan Provinces toward higher-elevation areas in western Sichuan and the adjacent regions of the Tibet Autonomous Region. Among the scenarios examined, the SSP5-8.5-2070s scenario shows the greatest migration distance, reaching 492.49 km, with the centroid shifting from Sichuan Province into the Tibet Autonomous Region. This pattern indicates a consistent tendency for the forest musk deer to track suitable climatic conditions by moving toward higher-elevation regions under ongoing climate change.

## 4. Discussion

The MaxEnt model is a widely applied predictive approach that infers species distributions based on known occurrence records and environmental variables under a presence-only framework [34]. In this study, habitat suitability for the forest musk deer was assessed by integrating environmental predictors with occurrence data derived from field surveys, literature records, and authoritative databases such as GBIF and the IUCN. The resulting AUC values (0.899 and 0.940) indicate strong model performance, suggesting that the selected environmental and socioeconomic predictors effectively captured major gradients associated with the species’ distribution. Under current climatic conditions, the predicted distribution encompassed nearly all known occurrence locations while also identifying extensive areas of potential suitable habitat. Collectively, these results suggest that the model provides a reliable representation of the forest musk deer’s distribution across China.

The forest musk deer has been widely reported to be closely associated with forested environments, showing a preference for coniferous forests, broadleaf forests, and mixed conifer–broadleaf forests. Evidence further suggests that habitat use varies seasonally. Previous field-based studies and telemetry observations indicate that mixed conifer–broadleaf forests are preferentially used during spring and autumn, coniferous forests are more frequently occupied in summer, and sun-exposed forest areas are selected in winter. These seasonal shifts likely reflect changes in food availability, microclimatic conditions, and shelter requirements [35]. In this context, the substantial contribution of NDVI identified in the present study is ecologically consistent with these documented habitat use patterns. As an integrative indicator of vegetation productivity and canopy structure, NDVI is closely related to the availability of forage resources and cover for forest-dwelling herbivores. Similar associations between vegetation indices and habitat suitability have been reported for musk oxen and other cervid species, underscoring the importance of vegetation dynamics in shaping their spatial distributions [36].

Socioeconomic variables, such as gross domestic product (GDP) and population density, were also associated with patterns of habitat suitability. However, these variables should not be interpreted as indicating direct causal effects; rather, they likely reflect broader gradients of land use intensity and landscape transformation. Areas characterized by higher economic activity and population density are often accompanied by infrastructure expansion, increased accessibility, and alterations in vegetation structure [37]. Given the pronounced territorial behavior of forest musk deer, with individuals typically occupying exclusive home ranges [38], such landscape modifications may disrupt habitat continuity and reduce the availability of suitable core habitats. Accordingly, the observed relationships are better understood as spatial associations between land use intensity and habitat suitability, rather than as evidence of negative impacts arising solely from human presence.

Importantly, the ecological effects of human activities are not uniformly negative and can vary substantially depending on hunting intensity, management practices, and spatial context [9]. In many regions, regulated hunting, habitat restoration initiatives, forest management programs, and captive breeding efforts have contributed positively to the stabilization or recovery of threatened wildlife populations [11]. In China, long-standing hunting bans, the establishment of nature reserves, and large-scale afforestation programs have, to some extent, reduced direct resource extraction and improved habitat conditions in certain areas.

In addition, agricultural and semi-natural landscapes can serve as supplementary habitats or movement corridors for certain wildlife species, particularly within heterogeneous landscapes. These examples underscore that human–wildlife interactions involve complex trade-offs. Biodiversity outcomes are therefore shaped not simply by the presence of humans, but by the quality of governance, land use planning, and adaptive management strategies [11].

Climatic variables, such as temperature seasonality (BIO4) and annual precipitation (BIO12), may further influence habitat suitability indirectly by shaping patterns of human land use. Regions characterized by climatic conditions favorable for agriculture or urban development often support higher GDP levels and greater population densities. These socioeconomic patterns can intensify land use conversion and reduce the availability of suitable habitats for forest musk deer. Consequently, the spatial patterns of habitat suitability identified in this study likely reflect the combined effects of intrinsic environmental suitability and the species’ avoidance of heavily modified landscapes. This highlights the interactive and co-determining roles of climatic and anthropogenic drivers in shaping species distributions.

Previous studies have found that elevation significantly influences the distribution of the forest musk deer, with populations showing a preference for habitats at elevations between 1500 and 1900 m [39]. The present study indicates that elevations of 1500 to 3000 m constitute suitable altitudinal range for the species, encompassing the findings of earlier research. Higher elevations are subject to lower level of anthropogenic disturbance, which may increase the probability of forest musk deer occurrence within these altitudinal zones.

Precipitation and temperature not only directly affect the survival of the forest musk deer but also influence, to some extent, its food and water sources. Studies have shown that food and water availability are determinant factors for habitat selection in wildlife [40]. The probability of forest musk deer occurrence initially shows an increase followed by a decline with rising annual precipitation (BIO12), with optimal condition occurrences at approximately between 750 and 1500 mm [17]. Adequate precipitation supports both water and vegetation availability for the forest musk deer, thereby sustain the necessary resources on which forest musk deer depend. Temperatures between 27 °C and 30 °C appear to be the most favorable for the forest musk deer survival and development and promote the growth of herbaceous and shrub plants in mixed coniferous–broadleaf forests. Together, these climatic conditions indirectly shape the spatial distribution of the species by influencing habitat quality and food availability.

Under current climatic conditions, the forest musk deer exhibits a highly fragmented pattern of suitable habitat, with highly suitable areas accounting for only 5.07% of the total suitable habitat. Compared with earlier studies, the present analysis provides a more constrained and refined estimate of suitable habitat extent. Previous research has demonstrated that limited occurrence records can lead to substantial overestimation of potential habitat ranges. By incorporating 1590 occurrence records with high spatial coverage across China, this study improves the robustness and spatial precision of habitat suitability estimates relative to earlier regional or data-limited assessments.

Our findings are broadly consistent with previous studies reporting a stronger negative response of forest musk deer habitats in southeastern China and greater resilience in southwestern mountainous regions. For example, Zhao et al. [41] identified pronounced habitat contraction in lowland and hilly areas of southeastern China, while Jiang et al. [42] reported an upward and northward shift in the suitable distribution of musk deer species under climate change. Building upon these studies, our research extends their conclusions by integrating updated occurrence data, anthropogenic disturbance factors, and CMIP6 climate projections at a national scale. This integrated framework allows for a more comprehensive assessment of how climate change and human activities jointly shape both current and future habitat suitability for the forest musk deer.

Notably, this study highlights the relative stability of suitable habitats in high-elevation regions of southwestern China, particularly along the border regions of Sichuan, Qinghai, and Tibet. These areas are characterized by complex topography, extensive forest cover, and comparatively lower levels of human disturbance, which may buffer the impacts of climatic warming. In contrast, suitable habitats in southeastern and coastal regions, dominated by plains and hills, are projected to undergo substantial contraction, likely reflecting greater climatic variability and intensified anthropogenic pressure.

The forest musk deer populations prefer habitats within coniferous forests, broadleaf forests, and mixed coniferous–broadleaf forests. Under future climate scenarios, the stable areas of suitable distribution for the forest musk deer are predominantly forested zones. Accordingly, we recommend continuously advancing forest conservation programs, restoring and protecting woodland areas, and reducing the expansions of human-activity such as construction and agriculture to safeguard the habitat for the forest musk deer. Future research should focus on establishing long-term monitoring frameworks in regions identified as both habitat contraction zones and potential climatic refugia. In particular, systematic population and habitat monitoring along elevational gradients in southwestern China would facilitate early detection of distributional shifts.

From a management perspective, the results emphasize the need for integrative conservation strategies that account for both environmental dynamics and socioeconomic realities. Rather than viewing conservation and development as opposing objectives, spatial planning approaches that incorporate habitat suitability modeling can help identify priority areas for protection, ecological corridors, and zones for sustainable land use.

In regions projected to remain suitable under future climates, particularly high-elevation forest areas, maintaining habitat connectivity and minimizing fragmentation will be critical. In lower-elevation regions, targeted restoration, landscape heterogeneity, and adaptive management may help mitigate habitat loss while supporting human livelihoods. Such evidence-based approaches offer a pathway toward reconciling biodiversity conservation with long-term socioeconomic development.

## 5. Conclusions

This study utilized twenty environmental variables and five anthropogenic disturbance factors to conduct a habitat suitability analysis for the forest musk deer population based on the MaxEnt model. The results indicate that the primary natural environmental factors influencing the distribution of the forest musk deer are annual precipitation, temperature, and elevation, while key anthropogenic factors include the Normalized Difference Vegetation Index (NDVI), Gross Domestic Product (GDP), and population density. Under current climatic conditions, the forest musk deer is primarily distributed across several regions in China, including Tibet, Sichuan, Yunnan, and Guangxi. Under future climate scenarios, however, the model predicts a noticeable shift in suitable habitats, with distributions moving toward higher latitudes and elevations. These projected shifts underscore the need to establish or expand protected areas in montane and northern regions, and to develop adaptive conservation strategies that account for climate-driven habitat displacement. Suitable areas in coastal regions are expected to contract substantially. These findings provide a scientific basis for the management and conservation of forest musk deer habitats in China.

## Figures and Tables

**Table 1:** The source of the environmental factors used for modeling.

Factor Type		Variables	Description
Natural factor	Climatic factors	BIO3	Isothermality (BIO2/BIO7) (×100)
		BIO4	Temperature seasonality
		BIO9	Mean temperature of driest quarter
		BIO12	Annual precipitation
		BIO14	Precipitation of driest month
	Topographic factors	DEM	Digital Elevation Model
Natural factor	Vegetation factors	NDVI	Normalized difference vegetation index (%)
	Human interference factors	Distance to road	Distance to road (m)
		PD	Population density
		PA	Protected area
		GDP	Gross domestic product

**Table 2:** Contribution value of different predictor variables in forest musk deer habitat suitability.

Variable	Percent Contribution	Cumulative Percent Contribution
BIO12	43.5	43.5
BIO4	32.3	75.8
DEM	13.3	89.1
BIO3	4.8	93.9
BIO9	3.2	97.1
BIO14	2.9	100

**Table 3:** Changes in suitable habitat area of the forest musk deer under future climate scenarios, including habitat loss, gain, and persistence relative to current conditions.

Climate Scenarios	Range Contraction (×10^4^ km^2^)	Range Expansion (×10^4^ km^2^)	No Change (×10^4^ km^2^)
SSP1-2.6-2030s	58.49	20.77	50.91
SSP1-2.6-2050s	55.1	21.11	54.32
SSP1-2.6-2070s	59.41	20.14	50
SSP5-8.5-2030s	45.35	25.29	64.05
SSP5-8.5-2050s	58.87	23.52	50.53
SSP5-8.5-2070s	65.73	28.85	43.69

## Data Availability

The data presented in this study are available in the manuscript.
